# Wilms' Tumor: Distribution, Demographics, and Outcomes in Sana'a City, Yemen

**DOI:** 10.7759/cureus.112639

**Published:** 2026-07-14

**Authors:** Aziz A Shamsan, Khaled M Alkohlany, Majdi Alshami, Mahmood Shaif, Nasr Al-Hakimi, Abdullah S Al-Sakkaf, Wael Alsagaf, Ahmed Farei, Nasser Albaddai

**Affiliations:** 1 Urology Department, General Military Hospital, Sana'a, YEM; 2 Urology Department, Sana'a University, Sana'a, YEM; 3 Surgical Department, 21 September University of Medical and Applied Sciences, Sana'a, YEM; 4 Surgical Department, Faculty of Medicine and Health Sciences, University of Saba Region, Marib, YEM; 5 Surgical Department, Faculty of Medicine and Health Sciences, Albaydha University, Albaydha, YEM

**Keywords:** cog staging, nephroblastoma, pediatric oncology, survival outcomes, wilms’ tumor, yemen

## Abstract

Background

Wilms' tumor (WT) is the most common pediatric renal malignancy (~90% of childhood kidney tumors). Survival exceeds 90% in high-income countries, but data from low-resource settings are limited. This study describes the demographic distribution, clinical presentation, management patterns, and short-term outcomes of pediatric WT in Sana'a, Yemen.

Methods

This prospective study (January 2021-December 2022) included children ≤15 years with Wilms' tumor diagnosed by clinical assessment, radiological imaging (abdominal ultrasound and contrast-enhanced CT and chest CT to assess local tumor extension and the presence of distant metastases), and histopathological confirmation following surgical resection. Demographic, clinical, radiological, surgical, and pathological data were prospectively collected using a standardized case report form. Tumors were staged according to the Children's Oncology Group (COG) criteria, and treatment modalities and short-term outcomes were evaluated.

Results

Fourteen pediatric WT patients (n = 14) were identified, with male predominance (71.4%) and a mean age of 3.6 ± 3.5 years. Abdominal mass was the most common presentation (85.7%). By COG staging: Stage I (35.7%), Stage II (50.0%), Stage III (14.3%); all had favorable histology (100%). Preoperative chemotherapy was given to 28.6%, all underwent surgical resection (100%), postoperative chemotherapy to 21.4%, and none received radiotherapy. At a median follow-up of 15 months (range 7-22), complete remission occurred in 92.9% (13/14), while one patient (7.1%) relapsed and died at 18 months.

Conclusion

Pediatric WT in this Yemeni cohort presented at an early stage with favorable histology; despite limited chemotherapy use and the absence of radiotherapy, short-term remission rates were favorable. Longer follow-up and improved multimodal therapy are needed.

## Introduction

Wilms' tumor (WT), or nephroblastoma, accounts for over 90% of pediatric renal malignancies and represents approximately 3.2-11.1% of childhood cancers worldwide, with marked epidemiologic variation between regions and ethnic groups. The age-standardized incidence rate (ASR) ranges from 4.1 to 5.4 per million in Asian populations to 9.1-9.8 per million in North America and Europe, with the highest rates among Black populations (10.9 per million). In children under five, WT predominates, peaking between two and three years of age [1-3].

WT arises from remnants of the metanephric blastema, reflecting abnormal persistence of embryonic renal precursor cells. While most cases are sporadic, WT may be associated with congenital anomaly syndromes such as Wilms' tumor, Aniridia, Genitourinary anomalies, and mental Retardation (WAGR), Denys-Drash, and Beckwith-Wiedemann. At the molecular level, loss of heterozygosity (LOH) at chromosomes 1p and 16q and gain of chromosome 1q have been linked to poorer outcomes in favorable-histology WT. Other reported alterations include LOH at 4q and 14q, MYCN amplification, and changes at 7p, although these markers are not yet standard in clinical prognostic models [4-7].

Treatment success in high-income countries (HICs) exceeds 90% overall survival (OS), largely due to structured, multidisciplinary protocols from major cooperative groups. The Children's Oncology Group (COG) favors upfront nephrectomy followed by adjuvant therapy, whereas the International Society of Pediatric Oncology (SIOP) advocates preoperative chemotherapy to reduce tumor size before surgery. Despite differences in sequencing, both approaches yield comparable long-term survival, highlighting the effectiveness of risk-adapted strategies and the importance of minimizing long-term treatment toxicity [8-10].

In low- and middle-income countries (LMICs), survival remains significantly lower-often 25-53%-due to delayed presentation, advanced stage at diagnosis, limited access to imaging and pathology services, treatment abandonment, and inconsistent delivery of chemotherapy or radiotherapy. In some sub-Saharan and Central Asian series, 60-80% of patients present with stage III or IV disease, and both pathology review and radiotherapy are often incomplete or unavailable [11-13].

To our knowledge, published data on pediatric Wilms' tumor in Yemen remain extremely limited. This prospective study aims to address this gap by investigating the epidemiological characteristics of WT and contributing to improving the prognosis and quality of life for children with WT in our country. To describe the demographic distribution, diagnostic methods, clinical presentation, management patterns, and short-term outcomes of pediatric Wilms' tumor in Sana'a city.

## Materials and methods

Study design and setting

We conducted a prospective, multicenter cohort study across nine hospitals in Sana'a City, Yemen, including four teaching governmental hospitals and five additional governmental and private hospitals providing pediatric surgical care for children with Wilms' tumor, between January 2021 and December 2022.

Patients

Children aged ≤15 years with histologically confirmed Wilms' tumor, verified by local pathology review when available, were included in the study. Eligible participants were enrolled after obtaining informed consent from a parent or legal guardian. Patients with non-renal tumors, those without histopathologic confirmation, or with incomplete diagnostic records were excluded.

Data collection

Data were obtained using a standardized case report form and included demographic information (age, sex, and family history of urogenital tumors) and clinical characteristics such as presenting complaints, symptom duration, abdominal mass size, hematuria, and hypertension. Imaging studies comprised abdominal ultrasound, contrast-enhanced CT scan, and chest CT to assess local tumor extension and the presence of distant metastases. Surgical findings recorded included tumor laterality, size, invasion of surrounding structures, and the number of lymph nodes removed. Histopathological evaluation was performed on surgical resection specimens, and tumors were classified according to the Children's Oncology Group (COG) criteria into favorable-histology and anaplastic subtypes; staging was assigned using the COG staging system [14]. Treatment details captured the type of surgical intervention (nephrectomy or excisional biopsy). Chemotherapy, when indicated, was administered by the pediatric oncology teams according to institutional protocols. The study recorded only the timing of chemotherapy (preoperative or postoperative), not the specific regimens, dosages, or duration. Radiotherapy was administered only when clinically indicated; however, none of the patient's received radiotherapy. Outcomes documented were treatment completion, abandonment, relapse, and two-year event-free survival and overall survival.

Follow-up

Patients were reviewed weekly during the early postoperative period and every three months thereafter. Follow-up included clinical assessment and abdominal ultrasonography or CT scan as indicated, with chest CT performed when recurrence or pulmonary metastasis was suspected.

Statistical analysis

Data were analyzed using SPSS version 28.0 (IBM Corp., Armonk, New York, USA). Continuous variables were summarized as means with standard deviation (SD), and categorical variables as frequencies and percentages.

Ethical considerations

The study was approved by the scientific committee of the Arab Board of Urology in Sana'a City, Yemen, Approval No. GMH-IRB-2024-WT-01. All participants' data were anonymized to ensure confidentiality, and informed written consent was obtained from parents or guardians.

## Results

During the study period, a total of 14 pediatric patients were diagnosed with Wilms' tumor. The majority were male (n = 10, 71.4%), with females accounting for 28.6% (n = 4). Half of the cohort (n = 7, 50.0%) were aged between 0.5 and 2 years, followed by 3-4 years (n = 4, 28.6%) and 5-18 years (n = 3, 21.4%). The mean age was 3.6 ± 3.5 years, ranging from 7 months to 13 years (Table 1).

**Table 1:** Demographic characteristics of patients with Wilms' tumor.

Variable	Category	n	%
Gender	Male	10	71.4
	Female	4	28.6
Age group	0.5-2 years	7	50.0
	3-4 years	4	28.6
	5-18 years	3	21.4

**Figure 1:**
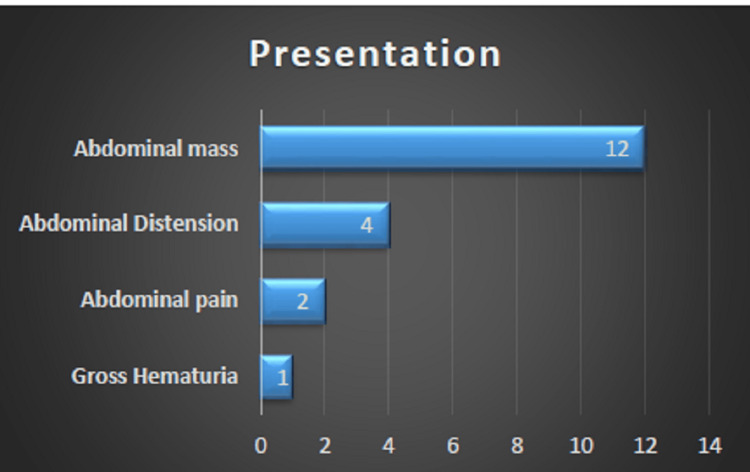
Presenting symptoms among cases.

The figure summarizes the frequency and percentage of presenting symptoms observed at diagnosis. Patients could present with more than one symptom; therefore, percentages may exceed 100% when combined. The most common presenting symptom was abdominal mass (n = 12, 85.7%), followed by abdominal distension (n = 4, 28.6%) and abdominal pain (n = 2, 14.3%) (Figure 1).

Radiological evaluation by CT scan revealed that the majority had right-sided tumors (n = 9, 64.3%), while five patients (35.7%) had left-sided tumors; none had bilateral disease. Tumor volume exceeded 500 mL in three patients (21.4%), while 11 (78.6%) had tumors smaller than 500 mL. All tumors were solid in consistency. Local invasion was present in four cases (28.6%), retroperitoneal lymph node metastasis in two cases (14.3%), and distant metastasis to the lungs in one case (7.1%) (Table 2).

**Table 2:** Radiological findings of Wilms' tumor cases.

Parameter	Category	n	%
Tumor site	Right	9	64.3
	Left	5	35.7
Tumor volume	<500 mL	11	78.6
	>500 mL	3	21.4
Consistency	Solid	14	100.0
Local invasion	Present	4	28.6
	Absent	10	71.4
Retroperitoneal lymph node (LN) metastasis	Present	2	14.3
	Absent	12	85.7
Distant metastasis	Present	1	7.1
	Absent	13	92.9

All patients (n = 14) underwent surgical excision as the primary treatment modality. Preoperative chemotherapy was administered in four patients (28.6%) due to factors such as large tumor size, suspected local invasion, or the need to facilitate safe resection. Postoperative chemotherapy was given to three patients (21.4%), primarily based on pathological staging and the presence of higher-risk features. None of the patients (n = 0, 0%) received postoperative radiotherapy, as no clear indications for its use were identified within the cohort (Table 3).

**Table 3:** Treatment modalities for Wilms' tumor patients.

Treatment modality	n	%
Preoperative chemotherapy	4	28.6
Surgical excision	14	100.0
Postoperative chemotherapy	3	21.4
Postoperative radiotherapy	0	0

Histopathological evaluation of all resected specimens was performed according to the Children’s Oncology Group (COG) classification. All tumors demonstrated favorable histology, with no cases of focal or diffuse anaplasia identified. Regarding histological subtype, all tumors exhibited a mixed pattern composed of variable proportions of epithelial, blastemal, and stromal elements. Tumor staging included Stage I (n = 5), Stage II (n = 7), and Stage III (n = 2), with Stage III cases receiving both preoperative and postoperative chemotherapy. Epithelial components ranged from 3% to 70%, blastemal components from 4% to 90%, and tumor necrosis from 0% to 90%. High necrotic fractions (>80%) were observed in several cases, particularly those with lower epithelial and blastemal proportions, while elevated blastemal predominance was noted in selected cases, including one Stage III tumor with 90% blastemal composition (see Table 4).

**Table 4:** Histopathological features of Wilms' tumor cases classified according to COG criteria. COG: Children's Oncology Group.

Case no.	COG stage	Histological subtype	Epithelial (%)	Blastemal (%)	Tumor necrosis (%)
1	II	Mixed	70	10	20
2	I	Mixed	6	4	90
3	III (Pre+Post-operative chemotherapy)	Mixed	10	90	0
4	III (Pre+Post-operative chemotherapy)	Mixed	70	10	20
5	II	Mixed	70	10	20
6	I	Mixed	6	4	90
7	II (Post-operative chemotherapy)	Mixed	50	30	20
8	II	Mixed	5	6	89
9	II	Mixed	3	12	85
10	I	Mixed	20	5	75
11	I	Mixed	10	60	30
12	I	Mixed	30	60	10
13	II (Preoperative chemotherapy)	Mixed	70	20	10
14	II (Preoperative chemotherapy)	Mixed	30	60	10

**Figure 2:**
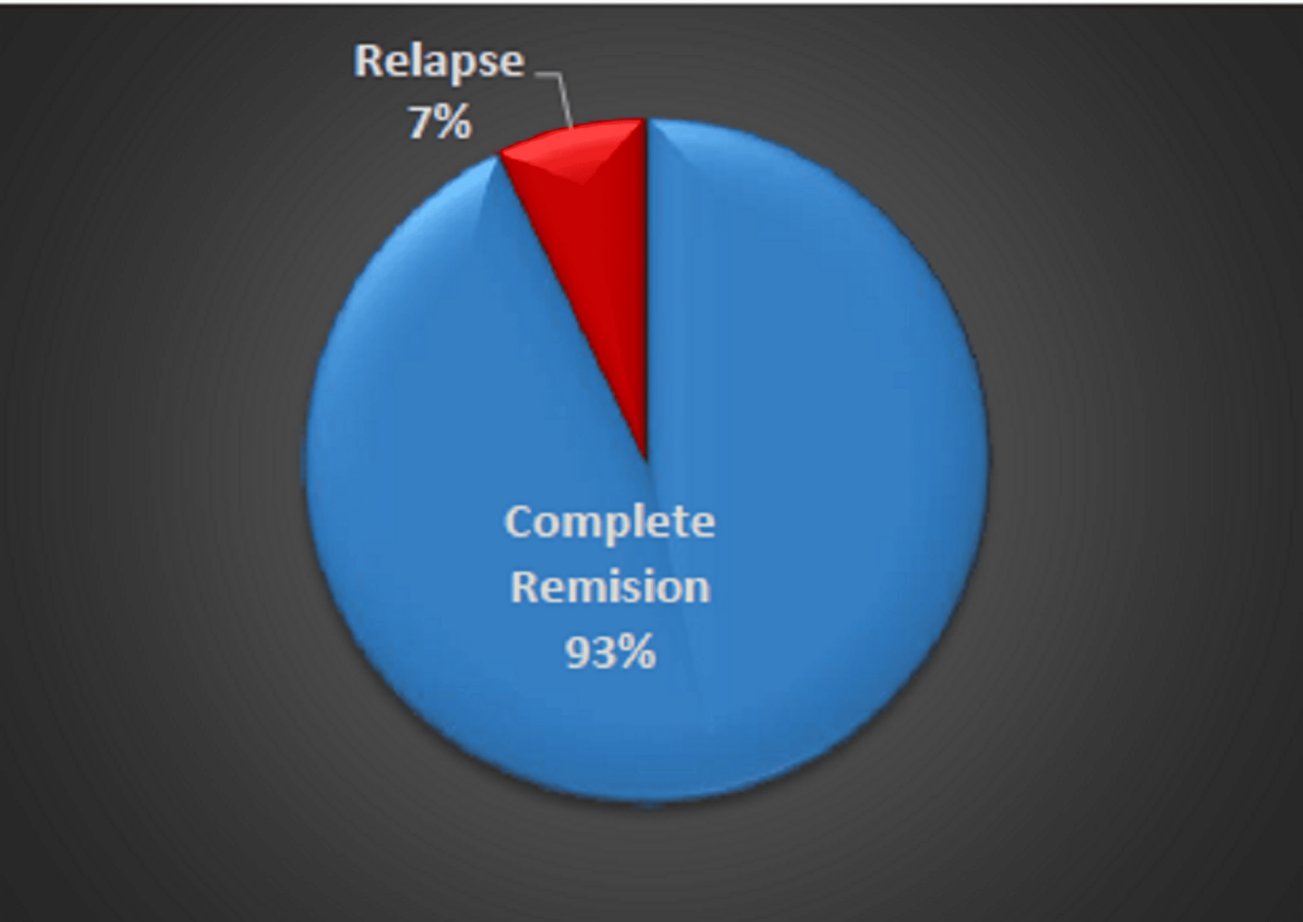
Final outcome.

The figure summarizes short-term outcomes during the follow-up period (7-22 months), including complete remission, relapse, and mortality among the study cohort. Follow-up data were available for all patients. During follow-up (7-22 months), 13 children (92.9%) achieved complete remission without evidence of recurrence on clinical and radiological assessment. One patient developed disease relapse during chemotherapy and subsequently died 18 months after surgery. Outcome assessment was based on scheduled clinical follow-up visits together with abdominal imaging and chest CT when clinically indicated (Figure 2).

## Discussion

In this prospective cohort of 14 pediatric patients with Wilms' tumor in Sana'a City, Yemen, the observed male predominance (71.4%) and mean age of 3.6 years are broadly consistent with global epidemiological patterns [1,2], although large datasets from North America and Europe have shown a nearly equal sex distribution [9]. Reports from sub-Saharan Africa and the Middle East have described male predominance ranging from 55% to 70% [3,15]. The apparent gender disparity in our cohort may reflect sampling variation related to the small sample size rather than true biological or cultural differences.

The most common presenting symptom was an abdominal mass (85.7%), consistent with regional and international data where abdominal swelling is the predominant presentation in 80-95% of cases [3,11,12]. The absence of hematuria in our cohort contrasts with reports describing rates up to 25% [16], which may reflect tumor characteristics or under-recognition in younger children.

Radiologically, right-sided tumors predominated (64.3%), consistent with some African and Asian reports [15,12], although most literature indicates no consistent laterality preference [8,13]. Only 21.4% of patients had tumor volumes exceeding 500 mL, suggesting a relatively earlier presentation compared to Cunningham et al., who reported a >50% large-tumor rate in delayed-diagnosis settings [3]. Rates of local invasion (28.6%) and nodal metastasis (14.3%) were within those reported in African series (20-40% and 10-20%, respectively) [15,12]. Only one patient (7.1%) had distant metastasis, markedly lower than the 15-30% reported in high-stage presentations in LMICs [3,11]. These findings may reflect the small sample size, referral patterns to tertiary centers in Sana'a, selection bias, and the short follow-up duration rather than the true population-based epidemiology or healthcare performance in Yemen.

With respect to treatment, all patients (n = 14) underwent surgical excision as the primary treatment modality. Preoperative chemotherapy was administered in 28.6% of patients, primarily in those with large or potentially unresectable tumors to facilitate safer resection, rather than being applied universally as in SIOP-based protocols [9,10]. Postoperative chemotherapy was given to 21.4% of patients, mainly those with higher-stage disease or additional risk factors, while the majority (71.4%) were managed with surgery alone, consistent with a selective, risk-adapted approach for low-risk, favorable histology tumors. In contrast, adherence to complete multimodal therapy in high-income countries exceeds 95% [8,10], contributing to survival rates above 90%. The absence of radiotherapy in all patients reflects a limitation in treatment capacity, as radiotherapy is indicated in selected higher-risk cases. Treatment deviations and resource constraints in low- and middle-income settings have been associated with poorer outcomes [3,17].

Histopathological staging revealed a predominance of Stage I-II disease (85.7%), with no Stage IV or V cases. This distribution differs from other LMIC reports, where 40-70% present with stage III-V disease [11,15]. This difference may partially explain the favorable short-term outcomes observed in our study and is likely attributable to the small sample size, referral patterns, and potential selection bias rather than true differences in disease biology or healthcare quality. All cases were favorable histology, which is consistent with the fact that unfavorable histology occurs in only 5-10% of WT globally [8], though its detection in LMICs may be limited by pathology constraints [17].

Our survival rate of 92.9% over a median follow-up of up to 22 months is encouraging and exceeds typical LMIC survival rates (25-60%) [3,15]. However, this finding should be interpreted cautiously due to the short follow-up duration, small sample size, and the skewed stage distribution toward lower-stage disease. Only one patient experienced relapse and died, corresponding to a relapse rate of 7.1%, which is lower than typically reported rates of 15-20% [6,8].

While early-stage presentation and favorable short-term outcomes are promising, these findings are constrained by notable limitations: small sample, short follow-up limiting recurrence/survival assessments, heterogeneous surgical techniques and non-standardized chemotherapy, absent radiotherapy and selective perioperative chemo hindering COG/SIOP comparisons, unreported operative variables (lymph nodes, margins, organ resection) and molecular markers, and multicenter variability with referral bias. Addressing these gaps-compounded by limited multimodal protocols and variable chemotherapy-requires strengthened diagnostics, improved access to comprehensive care, and unified protocols in larger, longer-term multicenter studies.

## Conclusions

Pediatric Wilms' tumor in this Yemeni cohort showed early-stage predominance and uniformly favorable histology. Despite limited multimodal therapy (no radiotherapy and selective chemotherapy), short-term remission was high. However, small sample size, short follow-up, and treatment gaps highlight the need for unified protocols, radiotherapy access, and stronger pathology services to improve long-term outcomes.
